# A photosynthetic rate prediction model using improved RBF neural network

**DOI:** 10.1038/s41598-022-12932-9

**Published:** 2022-06-10

**Authors:** Liuru Pu, Yuanfang Li, Pan Gao, Haihui Zhang, Jin Hu

**Affiliations:** 1 College of Mechanical and Electronic Engineering, Northwest A&F University, Yangling, 712100 Shaanxi China; 2 Key Laboratory of Agricultural Internet of Things, Ministry of Agriculture, Yangling, 712100 Shaanxi China

**Keywords:** Light responses, Photosynthesis

## Abstract

A photosynthetic prediction rate model is a theoretical basis for light environmental regulation, and the existing photosynthetic rate prediction models are limited by low modeling speed and prediction accuracy. Therefore, this paper analyses effects of light quality on photosynthesis rate, and proposes a method based on Radial basis function (RBF) optimized by Quantum genetic algorithm (QGA) to establish photosynthetic rate prediction model. We selected "golden embryo^2^ formula 98-1F1" cucumber seedlings as experimental material and used LI-6800 to record the photosynthetic rates under different temperatures, light intensities and light quality. Experimental data is used to train and test the proposed model. The determinant coefficient of the model between the predicted and the measured values is 0.996, the straight slope of linear fitting is 1.000, and the straight intercept of linear fitting is 0.061. Moreover, the proposed method is compared with 6 artificial intelligence algorithms. The comparison results also validate that the proposed model has the highest accuracy compared with other algorithms.

## Introduction

Photosynthesis is the main method to accumulate the substance for plant. Light is a necessary condition for photosynthesis and one of the environmental factors that is inseparable for the plant life. It not only provides the energy for plant photosynthesis, but also acts as a source to control and adjust the morphology of growth process^1–3^. The temperature indirectly influences the photosynthesis by affecting the activity of some enzymes in the process of photosynthesis^4^. Light environment regulation has become the main source of improving the crop yield and quality, and photosynthetic rate prediction model as the basis of building light environment regulation theory has been widely studied^5,6^. The existing prediction models do not consider the influence of light quality on photosynthetic rate and have low accuracy. Therefore, it is essential to solve light environmental regulation by constructing high precision photosynthetic rate prediction model.

Artificial neural network is widely used as a new intelligent modeling method in multidimensional complex modeling^7,8^. Recently, several research studies have been conducted on the photosynthetic rate model. Various researchers have constructed neural network based simulated models of greenhouse photosynthesis that have improved the accuracy of the photosynthetic rate mode^9^. While some researchers have established photosynthetic rate prediction model used on back propagation neural network^10,11^. The forecasted and the measured value of its correlation coefficient values is 0.99, which effectively improves the fit of the model. However, the model does not consider the effect of light quality on photosynthetic rate.

Radial basis function (RBF) neural network not only has a strong nonlinear mapping capacity but also has high fault tolerance and robustness, which make it most suitable for solving nonlinear problems^12^. The RBF has the advantages of fast learning, function approximation, pattern recognition and classification^13,14^. However, it is difficult to determine the spread of the network. Quantum genetic algorithm (QGA) is the combination of quantum computation and genetic algorithm (GA). It is a newly developed probability evolutionary algorithm that not only has the global optimization ability but also has strong adaptive and strong commonality advantages^15^. It can quickly determine the global optimal solution over a large area of the neighborhood. Above researches provide a basis for the fusion of multiple algorithms to build a high-precision prediction model for photosynthesis.

Aiming at the problems above, the necessity to fuse light quality in photosynthetic rate prediction model is analyzed, and a method based on RBF optimized by QGA is proposed to establish photosynthetic rate prediction model in this paper. The paper also uses light quality, temperature and light intensity as input and photosynthetic rate as the output to construct the experimental sample set, and compares the different effects of the neural network modeling to verify the superiority of the model.

## Materials and methods

### Experimental materials

This experiment was conducted from 8th November to 8th December 2019 in department of mechanical and electronic engineering of Northwest Agriculture and Forestry University (north latitude 34°07′39'', longitude 107°59′50', elevation 648 m). The experimental cucumber variety was "golden embryo^2^ 98-1f1". The sprouting seed was soaked up in a petri dish and processed in low temperature when it was gonging to sprout. From 6th October to 6th November 2019, bowl seedling was used in a cave dish with 50 holes (540 mm*280 mm*50 mm) every day. The seedlings matrix for agriculture was special matrix and its nutrient contents were: organic mass fraction over 50%, humic acid mass fraction over 20%, and pH 5.5 ~ 6.5. From the seedling plant, the plant was placed in the MD1400 incubator (the Dutch sander company), and the light source in the incubator was made up of red (wavelength 630 nm) and blue (wavelength 460 nm) light bulbs. The temperature of the incubator was set to 25 °C, relative humidity was set to 60%, CO_2_ was set to 400 uL/L, and photo period was set to 14 h. Normal cultivation management was performed with no spraying of any pesticides and hormones.

This experiment used LI-6800 portable photosynthesis system produced by Li-COR company to measure the net photosynthetic rate under different nesting scenarios along with the temperature, CO_2_ concentration, the photon flux density and other environmental variants. Temperature controlling module was used to set the required temperature as 18, 21, 24, 27, and 30 °C. LED light source module was used to set 16 photon flux densities as 1600, 1400, 1200, 1000, 800, 600, 400, 200, 100, 50, 30, 20, 10, 5, and 0 μmol/(m^2^·s). Red /blue light mass ratio was set to 10, 20, 30, 40, 50, 60, 70, 80 and 90%, with mass ratio referring to the proportion of blue light to the total photon flux density. The CO_2_ injection module was used to set the CO_2_ volume ratio to 400 μL/L and the water control module was used to set the relative humidity to 60%. A cucumber healthy growth seedling with 2 leaves was randomly selected for the experiment, and in order to ensure the consistency of the experiment we select consistent growth seedling as the measuring objects. The timings for the measurements were selected 8:30–11:30 and 14:30–17:30 in order to avoid the effects of the plant siesta on the results of the experiment.

Before measuring the photosynthetic rate of leaves, the photon flux density was set to 1600 μmol/(m^2^·s) to make the leaves be light-adapted for 20 min. When the photosynthetic rate was stable, the photoresponse program of LI-6800 was started to record 15 net photosynthetic rate values corresponding to the 15 photon flux densities. The sampling interval of each photosynthetic rate was 300 s. In order to reduce the experimental error, the average values of the experimental conditions of each group were repeated three times.

The data of light quality temperature and light intensity were set as the inputs, the net photosynthetic rate was selected as the output. For the error data caused by machine, Dixon criterion is used to eliminate the gross error of 720 groups of measurement data. A total of 45 groups of data were deleted because of the gross error. To eliminate the impact of different dimensions on the training result of neural network, the sample data should be normalized to [0, 1] by linear function. The calculation formula of linear function is:

1 $$z^{^{\prime}} = \frac{{z - z_{\min } }}{{z_{\max } - z_{\min } }}$$

### Experimental method

The RBF neural network, as a three-layer forward network, can approximate any continuous function with arbitrary precision. Since the spread is the main factor affecting the RBF neural network, so the integration of multiple intelligent algorithms is the key to the optimization modeling of RBF neural network. The basic idea is firstly to use the intelligent optimization algorithm to optimize the spread of RBF neural network, and then build the model using the RBF neural network with the optimized spread.

QGA is a kind of newly developed probability evolutionary algorithm based on a quantum vector representation. A quantum bit probability amplitude is applied to chromosome encoding, a chromosome can be superimposed the expression of a number of States, and the use of quantum logic gates to achieve natural chromosome update operation optimization in order to achieve the goal. Thus, QGA has strong global optimization ability, strong adaptability and versatility to fast access the global optimal solution over a large area of the neighborhood. Therefore, QGA is used to optimize the spread of RBF neural network and then the QGA-RBF neural network is constructed.

**Figure 1:**
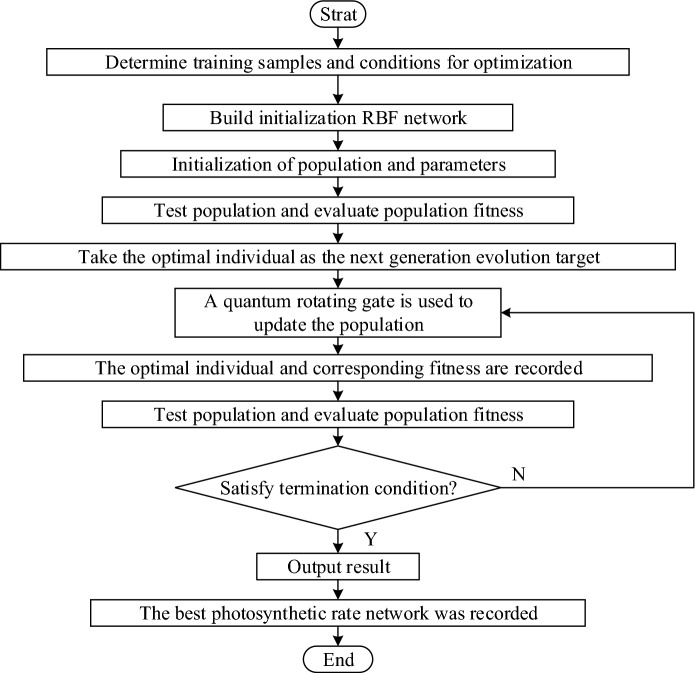
Flowchart of the QGA-RBF neural network.

This paper uses the QGA-RBF neural network to complete the photosynthetic model construction. Firstly, initial photosynthetic rate prediction model is established using the RBF network. Secondly, QGA is used to optimize the spread of RBF neural network. Finally, the optimal spread is assigned to the RBF neural network and the model of the photosynthetic rate prediction of QGA-RBF network is constructed. The modeling process is shown in Fig. 1.

### RBF neural network structure

**Figure 2:**
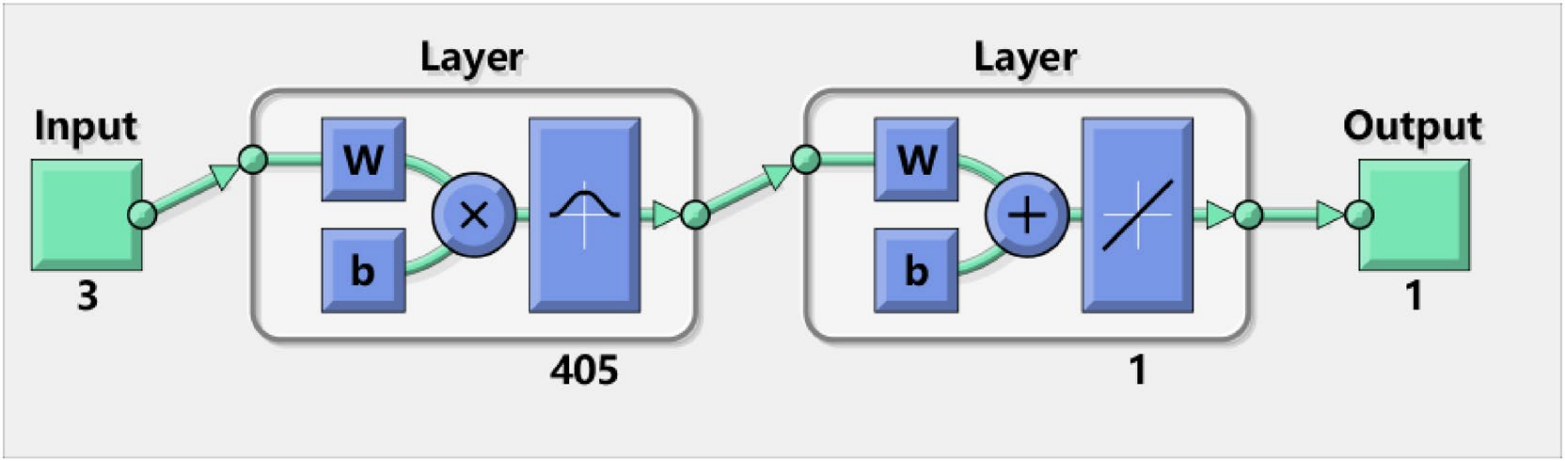
Typical RBF structure.

The RBF neural network is a type of forward neural network with structure similar to multi-layer forward network. It is a forward network of three layers^16,17^. The first layer is the input layer that consists of signal source nodes. The second layer is the hidden layer consisting of a variable number of neurons. The optimal number of neurons is determined by the training process. The second layer is composed of RBF neurons in the hidden layer. According to previous study, the number of neurons in the hidden layer should be set to be consistent with the number of samples, which is 405^18^. Each neuron consists of a radial basis function centered on a point with as many dimensions as there are predictor variables. The third layer is the output layer that responds to the input mode. The network structure is shown in Fig. 2.

The RBF neural network based on self-organizing data center is adopted. The algorithm mainly consists of two processes: the first process uses clustering method to determine the data centers and extend the constants of the radial basis functions of the hidden layer, which is a slow unsupervised learning process. The second process determines the weight of the output layer and is a fast supervised learning process.

- The basis function center $c$ is obtained based on the k- mean clustering method.
- Initialization: randomly select $h$ vectors that are different from each other as initial clustering centers $c_{i}$, $i = 1,2, \cdots ,h$.
- Calculate the Euclidean distance between the input sample and the cluster center:
  2 $$\left\| {X^{p} - c_{j} \left( k \right)} \right\|,\;\;p = 1,2, \ldots ,P;\;\;j = 1,2, \ldots ,M$$
- The input samples are grouped according to the nearest neighbor rule: the input sample is divided into the cluster of the least Euclidean distance.
- The adjustment of clustering center: after all input samples are grouped, the new clustering center is obtained by calculating the average of the samples in each cluster. Return to step (2) until the cluster center no longer changes and use it as the final clustering center of the RBF neural network.
- Solving variance $$\sigma_{i}$$.

The RBF neural networks usually use the Gauss function as the basis function and the variance is calculated as follows:

3 $$\sigma_{i} = \frac{{c_{\max } }}{{\sqrt {2h} }}\;\;\;i = 1,2, \ldots h$$

where $c_{\max }$ is the maximum distance between the selected centers.

c. Computing the weights between the hidden layer and the output layer.

The connection weights between the hidden layer and the output layer are calculated by the least square method:

4 $$\omega = \exp \left( {\frac{h}{{c_{\max }^{2} }}\left\| {x_{p} - c_{i} } \right\|} \right)\;\;\;{\kern 1pt} i = 1,2, \ldots ,h;\;\;p = 1,2, \ldots ,P$$

To Optimize the Spread of RBF Neural Network Based on QGA Algorithm.

The spread of RBF neural network directly determines the performance of the network. If it is too small, then the numbers of neuronal need to adapt the slow changes of the function. While, if it is too large, then the numbers of neuronal need to adapt the fast changes of the function. In order to test the impact of different spreads on the network, we chose different the spread ranging from 0.1 to 15.1 for photosynthetic rate prediction. As we can see in the Table 1, the spread have a main impact on the results.

**Table 1:** Effect of the spread on model results.

Number of the *spread*	Mean square error	Maximum absolute error μmol/(m^2^·s)	Mean absolute error %
0.1	44. 367	48. 238	0.847
1.1	51.768	44.634	0.840
2.1	10.928	32.216	0.990
3.1	5.529	19.532	0.991
4.1	1. 733	15.436	0.987
5.1	0. 865	7.554	0.975
6.1	0.739	5.467	0.664
7.1	0.467	4.072	0.989
8.1	0.358	3.074	0.984
9.1	0.356	2. 061	0.967
10.1	0.359	2.598	0.968
11.1	0.506	2.228	0.960
12.1	0.454	2.882	0.959
13.1	0.437	2.535	0.949
14.1	0.448	2.228	0.948
15.1	0.434	2.357	0.953

This paper uses QGA to optimize the spread of RBF neural network. According to Table 1, the initial scope [0.1 10] is selected as the splash range of the spread of the RBF neural network. Then to establish the mapping relation between the code string and the spread we perform binary string coding. In order to obtain the best spread of RBF neural network, the mean square error of the predicted values and the measured values of training is set as the fitness function, which is calculated as:

5 $$F = \frac{1}{P}\sum\nolimits_{i = 1}^{P} {\left( {pn_{d}^{^{\prime}} - pn_{o}^{^{\prime}} } \right)^{2} }$$

In above formula: $pn_{d}^{^{\prime}}$ and $pn_{o}^{^{\prime}}$ are the measured value and the predicted value of the photosynthetic rate, and $P$ is the number of test sets.

If it is unable to meet the stop condition of the network, the following operations are processed^19–21^:

- If the population is initialized as $$Q(t_{0} )$$, and all the genes $$\left( {\alpha^{\prime}_{i} ,\beta^{\prime}_{i} } \right)$$ of the entire chromosome in the population are initialized as $$\left( {{\raise0.7ex\hbox{$1$} \!\mathord{\left/ {\vphantom {1 {\sqrt 2 }}}\right.\kern-\nulldelimiterspace} \!\lower0.7ex\hbox{${\sqrt 2 }$}},{\raise0.7ex\hbox{$1$} \!\mathord{\left/ {\vphantom {1 {\sqrt 2 }}}\right.\kern-\nulldelimiterspace} \!\lower0.7ex\hbox{${\sqrt 2 }$}}} \right)$$, then this indicates that a chromosome represents the equal probability of all possible states:
  6 $$\left| {\psi_{qj}^{^{\prime}} } \right\rangle = \sum\limits_{k = 1}^{{2^{m} }} {\frac{1}{{\sqrt 2^{m} }}} \left| {S_{k} } \right\rangle$$
  where $S_{k}$ represents the $k$ state of the chromosome and it expressed in the form of a binary string of *m*($x_{1}$,$x_{2}$,…, $x_{m}$), where $x_{i}$ is 0 or 1.
- The fit measurement and the evaluation of the population. Individuals are randomly selected, and make an initial measurement of population in order to obtain a set of definite solutions $P\left( t \right) = \left( {p_{1}^{t} ,p_{2}^{t} , \ldots ,p_{n}^{t} } \right)$, where $p_{j}^{t}$ is the *i*th population *j*th solution expressed as m in length of binary string with 0 or 1 at one time that is selected according to the probability of quantum bit. For measuring process, an interval number in [0, 1] is randomly generated. If it is greater than the probability amplitude of the square, the measurement result value is 1, otherwise the measured value is 0. Then for fitness evaluation, record the best fitness individuals from this set of solutions as the evolution of the next target.
- Loop iteration to find the optimal solution. Firstly, the population is measured to obtain a set of determined solutions and then each fitness value is calculated. According to the evolution of the current goal and agreed-to adjustment strategy, quantum revolving door is used to adjust the individuals in the population to obtain the updated population. The current optimal solution is recorded and compared with the current target value, and if it is greater than the current target, use the new optimal solution as the target of the next iteration, otherwise use the current target. The optimal spread is given to the RBF neural network to build photosynthetic rate prediction model as:
  7 $$P_{h} = net\left( {pi,par,T} \right)$$

In above formula, $pi$ is the light quality, $par$ is the photon flux density and $T$ is the temperature.

## Results and discussion

### Analysis and verification of optimization effect

**Figure 3:**
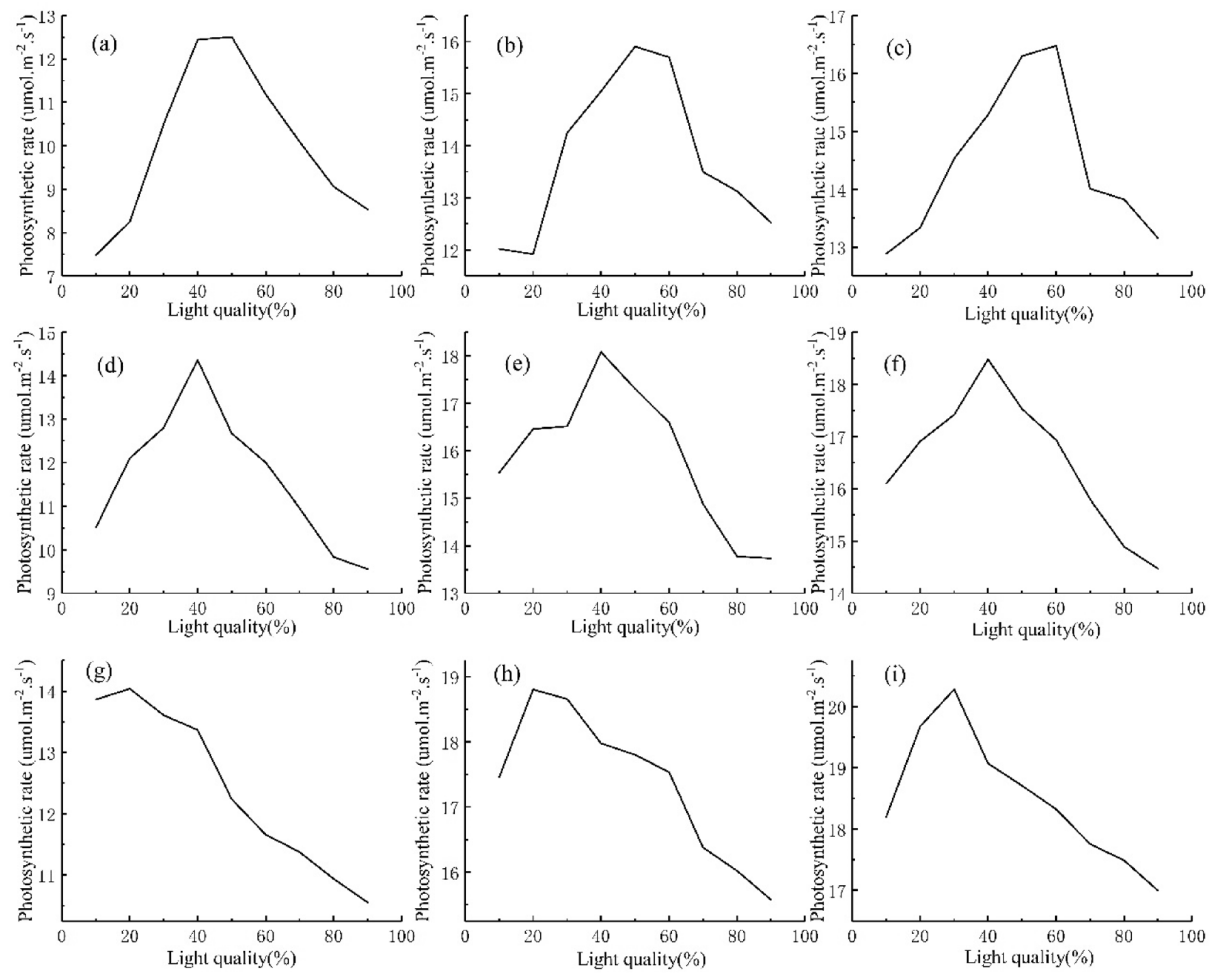
The change of photosynthetic rate with light quality: (**a**) At the temperature of 18 °C and the light intensity of 400 μmol/(m^2^·s); (**b**) At the temperature of 18 °C and the light intensity of 800 μmol/(m^2^·s); (**c**) At the temperature of 18 °C and the light intensity of 1200 μmol/(m^2^·s); (**d**) At the temperature of 24 °C and the light intensity of 400 μmol/(m^2^·s); (**e**) At the temperature of 24 °C and the light intensity of 400 μmol/(m^2^·s); (**f**) At the temperature of 24 °C and the light intensity of 400 μmol/(m^2^·s); (**g**) At the temperature of 18 °C and the light intensity of 400 μmol/(m^2^·s); (**h**) At the temperature of 18 °C and the light intensity of 400 μmol/(m^2^·s); (**i**) At the temperature of 18 °C and the light intensity of 400 μmol/(m^2^·s).

The existing models of photosynthetic rate only consider effects of light intensity on the photosynthetic rate and rarely analyze effects of light quality. For this reason, this paper chose the same temperature and light intensity to analyze the photosynthetic rate under different light qualities, as shown in Fig. 3. It can be seen from the figures that the photosynthetic rate is clearly different under the same temperature, light intensity and different light qualities, which highlights the significance of developing photosynthetic rate prediction model as one-dimensional input.

**Figure 4:**
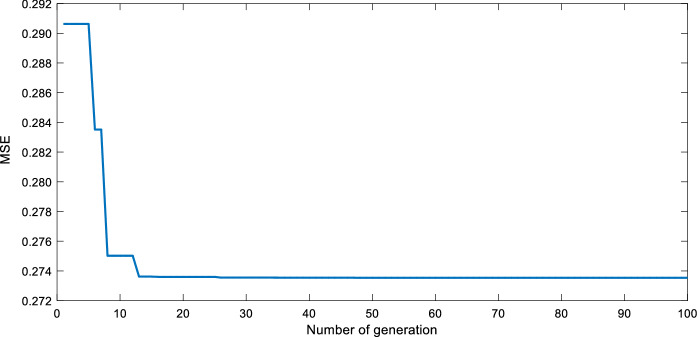
Variation curve of error.

In this paper, 80% of the experimental sample set is selected as training set, which is about 540 groups. While the remaining 20% is selected as the verification set, which is about 135 groups. In order to find the best spread, the residuals of the predicted and the expected values of the RBF neural network should be as small as possible, and the predicted value and the expected mean square error of training are selected as the target function. As illustrated in Fig. 4, the network evolves to 15 generations to find the best spread, the training error drops rapidly to the minimum mean square error (0.273) and the training process is neither volatile nor local plain area. Thus, the QGA-RBF neural network has good convergence.

### Model verification and analysis

**Figure 5:**
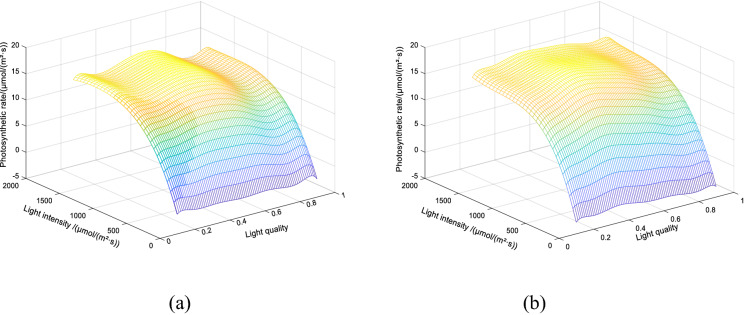
Photosynthetic rate prediction model at different temperatures. (**a**) At 21 °C; (**b**) at 27 °C.

The model can obtain photosynthetic rate under random temperature, light intensity and light quality. In order to test and verify the influence of light quality on the photosynthetic rate, the photosynthetic rate prediction model is mapped under 18 °C and 27 °C, as shown in Fig. 5. At different temperatures, the difference between the two models is obvious. The suitable light quality can improve the photosynthetic rate of the plant and can provide the model foundation for the adjustment of the light environment. Analyzing its reason: the light quality can raise the photosynthetic rate to some extent^18^. The reason for this raise is that the low and high temperature will affect the stomatal opening, enzyme activity, electron transfer rate, gas exchange parameters and photosynthetic pigment content. And blue light can not only improve the activity and the photosynthetic electron transport capability of light, but can also affect the stomatal opening and closing, and can drive the osmotic regulation mechanism of stomatal movement^22,23^. Therefore, in the case of low and high temperature, the improvement in the proportion of blue light can slow the effects of high and low temperature stress on crop photosynthesis and increase the photosynthetic rate. Two reaction centers of photosynthetic rate PSI and PSII focused on the red light wavelengths^19,20^. The red light quantity still dominates photosynthesis, and with the reduced degree of stress the blue light regulation demand lessen but red light demand is increased.

**Figure 6:**
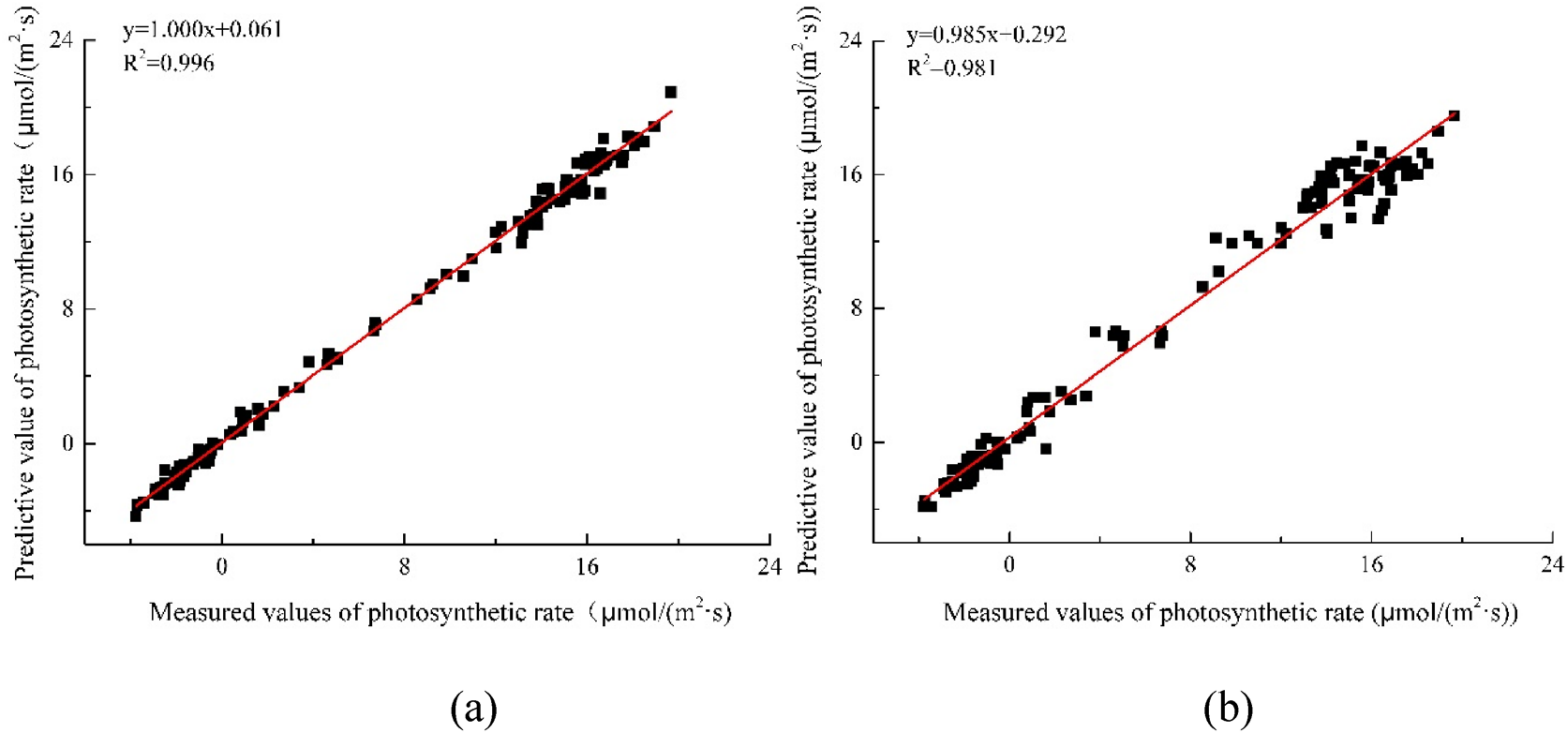
Correlation between the predicted and the measured values of photosynthetic rate. (**a**) Light quality added as input; (**b**) Without light quality as input. The red line is a linear fitting to the data.

In order to verify the effect of adding light quality as one-dimensional input, photosynthetic rate prediction model based on QGA-RBF neural network, this paper constructs two prediction model based on QGA-RBF neural network, one prediction model added light quality as one-dimensional input and anther not added light quality as a one-dimensional input, and use the same verification set test the performance of model. The results of the correlation analysis of the measured values of photosynthetic rate and the model predicted values are shown in Fig. 6.

Figure 6 shows that the photosynthetic rate model based on QGA-RBF neural network, with temperature, light quality and light intensity as inputs, has good fitting degree, which proves that the algorithm is suitable for complex system modeling of multidimensional input data model. When the input includes temperature, light intensity and light quality, the determinant coefficient of the model between the predicted and the measured values is 0.996, the straight slope of linear fitting is 1.000, and the straight intercept of linear fitting is 0.061.When the input only includes temperature and light intensity, the determinant coefficient of the model between the predicted and the measured values is 0.981, the slope of the fitting line is 0.985 and the intercept is 0.292. Obviously, fusion light quality can increase the fitting degree of the prediction model of photosynthetic rate.

**Figure 7:**
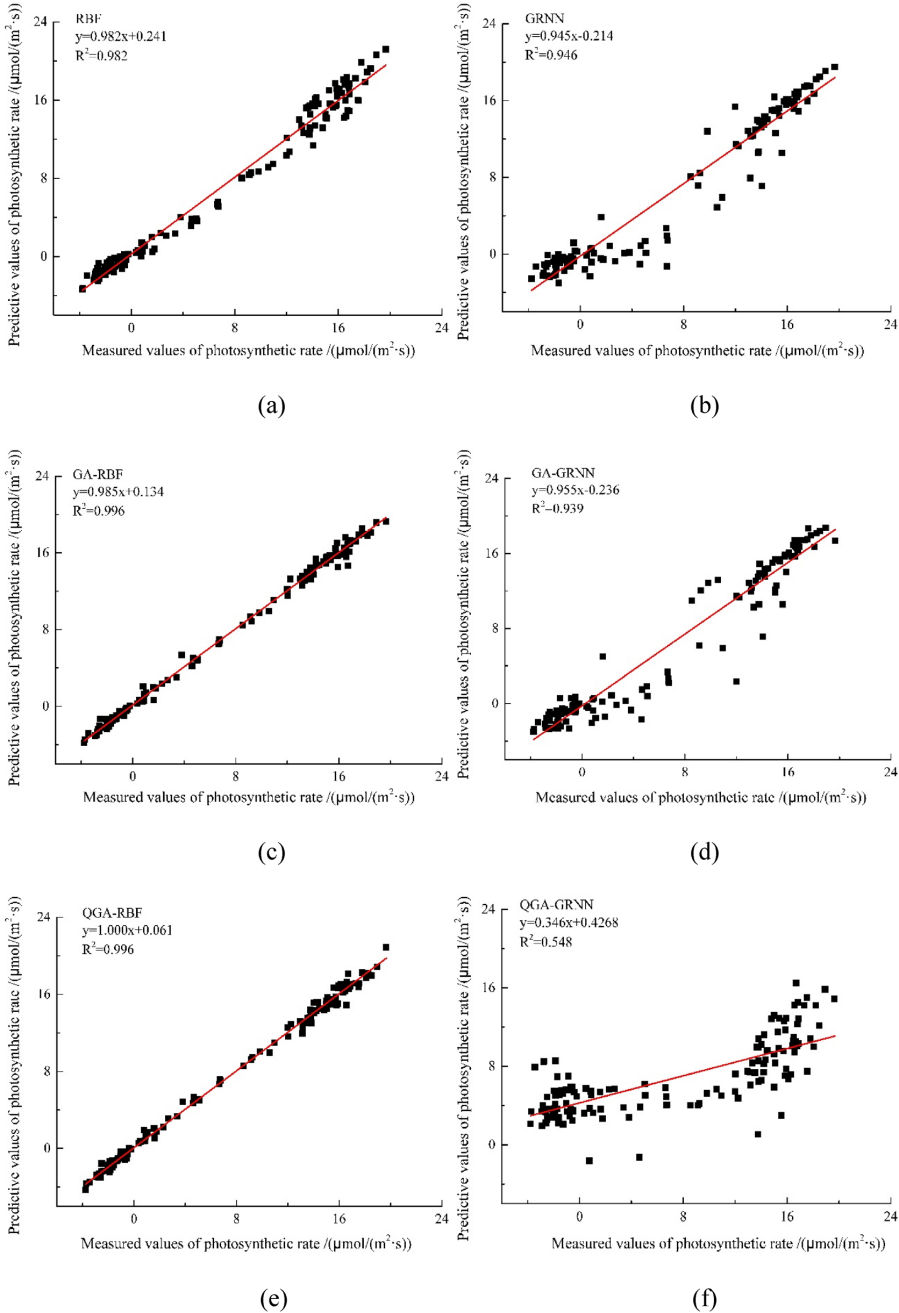
The performance of prediction model using different methods. (**a**) RBF (**b**) GRNN (**c**) QA-RBF (**d**) QA-GRNN (**e**) QGA-RBF (**f**) QGA-GRNN. The red line is a linear fitting to the data.

In order to further analyze the advantages of QGA-RBF network, the same training set and the verification set are used, and the six modeling approaches (RBF, GRNN, GA-RBF, GA-GRNN, QGA-RBF, QGA-GRNN). Similarly, QGA-GRNN is QGA algorithm optimized the spread of GRNN, GA-GRNN is GA algorithm optimized the spread of GRNN, and GA-RBF is GA algorithm^24^ optimized the spread of RBF. The performance of the six algorithms can be seen in Fig. 7, and analysis of the maximum absolute error and the mean absolute error for the six algorithms are summarized in Table 2.

**Table 2:** Evaluation index of prediction model based on different artificial intelligence algorithms.

Predicted models	RBF	GA-RBF	QGA-RBF	GRNN	GA-GRNN	QGA-GRNN
Maximum absolute error umol/(m^2^.s)	2.216	2.041	1.689	6.957	12.594	9.640
Mean absolute error umol/(m^2^.s)	0.874	0.342	0.337	1.371	1.342	0.874
Computing time s	0.077	0.103	0.040	0.028	0.021	0.036

As we can see in Fig. 7, the linear fitting formula of QGA-RBF is closer to y = x than other algorithms, and the Table 2 shows that QGA-RBF model in mean absolute error and the correlation coefficient is high indicating that there is a good correlation between the measured value and the predicted value, Moreover, the maximum absolute error of QGA-RBF is 1.689 μmol/(m^2^·s), and the mean absolute error of QGA-RBF is 0.337 μmol/(m^2^·s). The maximum absolute error and the mean absolute error are smaller than other algorithms. Table 2 shows that the accuracy of RBF algorithms is better than GRNN algorithms, and the computing time of QGA-RBF is the shortest in RBF algorithms. Various precision indices that QGA-RBF model performs very well and has a high prediction accuracy. The above analysis validates that QGA-RBF prediction model of photosynthetic rate has high accuracy and strong reliability.

## Conclusions

Under application requirements of environment light optimization control for protected agriculture, we consider the influence of light quality on crop photosynthetic rate and propose the photosynthetic rate modeling method based on QGA-RBF. The contributions of this paper are as follows:

1. This paper proposes a method of QGA-RBF to build photosynthetic rate model, which selects the best spread of RBF neural network by QGA. The determinant coefficient of QGA-RBF model between the predicted and the measured values is 0.996, very close to 1. The Maximum absolute error is 1.689 μmol/(m^2^·s) and the mean absolute error is 0.33 μmol/(m^2^·s), which is smaller than other algorithms. We can conclude that QGA-RBF prediction model of photosynthetic rate has high accuracy and strong reliability. Meantime, QGA-RBF provides a basis for building the photosynthetic rate model to verify the impact of light quality on the photosynthetic rate prediction.
2. This paper adopts QGA-GRNN neural network. Added light quality as one-dimensional input, the determinant coefficient of the model between the predicted and the measured values is 0.996, the straight slope of linear fitting is 1.000, and the straight intercept of linear fitting is 0.061. Not added light quality as a one-dimensional input, the determinant coefficient of the model between the predicted and the measured values is 0.981, the slope of the fitting line is 0.985 and the intercept is 0.292. It shows that fusion light quality is a necessary condition to set up a high photosynthetic rate prediction model.
